# Observations on the Powers and Effects of Cold as a Cause of Disease; with Some Remarks on the Best Means of Preventing Its Morbid Effects, &c.

**Published:** 1832-07

**Authors:** John Clendinning

**Affiliations:** Edinburgh and Oxford, Fellow and Censor of the Royal College of Physicians of London, and Physician to the Western Dispensary.


### COLD AS A CAUSE OF DISEASE.

*Observations on the Powers and Effects of Cold as a Cause of Disease; with some Remarks on the best Means of Preventing its Morbid Effects, &c.* By John Clendinning, M.D. of Edinburgh and Oxford, Fellow and Censor of the Royal College of Physicians of London, and Physician to the Western Dispensary.

(Continued from page 451 of the last volume.

*Respiratory Function.* The effect of even severe cold on the respiratory functions and organs is usually not very remarkable. *Hurry of respiration* is a familiar effect of the cold bath. Inhalation of intensely cold air has been followed by *sense of suffocation,* and even by a feeling of *laceration* in the chest. Fletcher, in his account of a Russian winter, *(Purchas's Pilgrimes,* vol. iii. b. 3,) states, that "when you pass out of a warm room into a cold, you shall feel sensibly your breathe waxe starke, and even stifeling with the cold as you draw it in or out." Maupertuis (*Figure de la Terre,* &c.) found the cold at Tornea, in December 1736, I think, so intense, that, "on opening a heated apartment, the external air rushing in, turned the vapour of the room into flakes of snow; and, on going out of doors, the air seemed to tear the chest." Even immediate death, from asphyxia apparently, has followed the sudden passage from a heated room into an extremely cold atmosphere; of which MACQUART has seen an example. If the chemical changes that take place in the lungs be, like several other animal processes, susceptible of increase and diminution from atmospherical agency, may not the suffering of asthmatics in cold weather be caused by suspension, at least in part, of the process of decarbonization? The influence of residence in a warm atmosphere, of the free use of alcohol, of mercurialization, &c. on the consumption of oxygen, proves that the chemical part of the arterializing process varies more or less with circumstances, and to a certain extent favors the supposition. No doubt, the additional load of fluids thrown on the pulmonary mucous membrane by the suspension of excretion in the skin must also be remembered, and is of the first importance in the theory of humid catarrhs, &c. If cold be capable, as above supposed, of suspending the chemical functions of the lungs, it is probable that sudden death from inspiration of cold air, of which BEAUPRÉ, MACQUART, BRAMBILLA, FLETCHER, and others have recorded examples, may, in some instances, be attributable to such an event.

*Animal Heat and Calorific Function.* The morbific effects of cold on animal heat and the calorific function are, 1st, Abstraction of Heat; 2d, Depression of the Power of evolving Heat; 3d, Reaction. Under refrigeration, the cutaneous warmth rapidly disappears, and an almost death-like coldness of the surface is readily produced. The viscera, however, and the blood circulating in the deeper-seated vessels, retain their normal heat long after the chilling of the surface. After having reduced my pulse twenty beats, and my heat in the axilla six or seven degrees, I found that the bladder had lost nothing of its natural warmth: its contents still gave ninety-eight or ninety-nine degrees of Fahrenheit. Reaction readily follows refrigeration, more particularly if the return of heat be favored by external warmth. That law of organic nature which resists disturbing causes, counteracts depressing agencies, expels noxious elements, supplies deficient conditions of life and health, is remarkably exemplified in the operation of cold in the living body. The glow experienced by persons in vigorous health after the first impression of coolness on the face, &c. in frosty weather, is a familiar illustration of this subject. The effect of exposing parts chilled by frost to the heat of warm water, or of a fire, is another instance. Such facts abound; but I have met with no proof of this recuperative, compensative energy so unequivocal as that furnished by the effect of immersion in cold water. In the situation of a healthy adult male, free from every excitement, moral and physical, fasting, just risen from bed, and immersed, with the least possible previous exertion, in a cold bath of still water, there can be but few sources of fallacy. In such circumstances, I have repeatedly found my heat (under the tongue,) to decline, not in the ratio of temperature or time of exposure, but irregularly, and so to speak, reluctantly, with frequent momentary rises and fluctuations, and those unaccompanied, so far as I could see, by any corresponding changes in the nervous or vascular systems. In the experiments of Dr. Currie, if they can be depended upon, results still more strikingly confirmatory of the existence of that organic power were obtained. Dr. Currie's observations on the effects of cold bathing furnish several instances in which not only was the heat of the subject found to decline irregularly, as above stated, but even after the lapse of some time, the thermometer was observed to rise again, so as, in the course of fifteen or twenty minutes, to reach within three or four degrees of the standard of health. Such a result I have myself never obtained as that last mentioned.

Protracted refrigeration depresses the calorific energy. The chemical processes are checked in the superficial and subcutaneous circulation, and the average temperature of the blood is lowered; the activity of the capillary vessels, and ultimately of the heart and great vessels, is diminished; the energy of the nervous functions is lessened; it is plain, therefore, that, from internal sources, the re-establishment of the normal heat after protracted refrigeration must be slow and gradual. Dr. Currie mentions that a man, whose heat before bathing was one hundred degrees, and after fourteen minutes immersion ninety-five degrees, and who was cooled down to eighty-eight degrees by three minutes' exposure after the bath to a north-east wind, had, at the end of an hour spent in a warm bed, and notwithstanding that he had had a draught of ale, attained to no higher degree than ninety-five degrees. In another experiment, the same person had not recovered his natural temperature (ninety-eight degrees) three hours after bathing and exposure to the wind, notwithstanding frictions, brandy and water, and the application of a bladder of hot water to his præcordia. I am aware that, in enumerating amongst the elementary causes of diseases of cold, reactive effort and depressed energy of the calorific function, I lay myself open to the charge of ascribing real sensible effects to mere abstractions and hypothetical entities. But I am no disciple of the mystic physiology of Montpellier; and I am well aware that, in pathological as in physiological ætiology, *vital properties*, *vital functions*, &c. are not substantive causes, but logical entities, or figures of speech. Since, however, it is uncertain what the agent or agents are that produce animal warmth, I have preferred using language involving no theory on the subject, and refer those who attribute animal heat to nervous or vascular action to what I shall hereafter say on the morbific effects of cold on the circulation, and on the nervous system.

*Vascular System.* The effects of cold on the vascular system have been much disputed. From the best consideration I have been able to give to the facts that have fallen under my own observation, or that I have met with in books, I have come to the conclusion that *Cold, in its immediate or direct operation on the heart and arteries, is purely sedative*, and that the phenomena owing to which various esteemed authors have, as I conceive, erroneously attributed to cold a stimulant power over these organs, have been referrible to the action of unobserved disturbing causes and sources of fallacy. I have made a considerable number of experiments with a view to the determination of this question: of these experiments I have generally myself been the subject. The form of application I have used in most instances has been the fresh water bath, at a temperature commonly twenty-four or twenty-five degrees above the freezing point; and the result of these trials many times repeated were uniformly confirmatory of the sedative action of cold. I usually entered the bath immediately after rising out of bed, and continued immersed until the supervention of rigors and brisk shivering, and found, for the invariable result of the experiment, a diminution of the frequency of the pulse of fifteen or twenty strokes, with some decrease of force also. I have some confidence in the result of these trials, because I know myself to have been perfectly free from any peculiarity of habit or susceptibility with respect to heat or cold, and because I took the greatest care to avoid every fallacy arising from inattention to diet or regimen, or to moral or physical agitation or exertion, or to bodily position before, during, and immediately after experiments. Some particulars of the results of those trials I published some years since, in an appendix to an English edition of a French work on the Medical History of Cold; and I have since that time (1825-6) met with nothing to alter my opinion on the subject.

The circumstances that have, as I think, misled various writers relative to the effects of cold on the circulating system are numerous. I shall point out some of the principal: 1st, Aversion to the bath is one cause; the influence of moral feeling over the heart's action is matter of hourly popular experience. If the subject of observation enter the cold medium reluctantly, we must not be surprised if, even after the lapse of some minutes, we can detect no diminution in the frequency or force of the heart's action. 2dly. Another cause is the extreme sensibility to changes of temperature observable over the whole cutaneous surface of some individuals. There is no viscus over which that most important organ, the skin, does not exert, under some circumstances, the most marked influence; the rousing, vivifying, otherwise stimulant, action of the cold affusion or cold douche in coma, insensibility, &c. from alcohol, opium, prussic acid, and other narcotic poisons, is well established; the restorative effect of sprinkling cold water on the face, neck, &c. in syncope is practically known to every fine lady. A third source of fallacy is disturbance of the respiratory function: on this subject I have the following observations in the volume formerly referred to. "In every instance in my experiments on the cold bath, I found the abdominal parietes yield to the water, so that much less than usual of the room necessary for the air inspired was obtained by pushing down the viscera, and consequently much unusual labour devolved on the elevators of the ribs. In some instances, the renewal of the preternatural state of respiration by leaving the bath was immediately, on sitting tranquilly for a little, followed by a fall of many strokes in the minute. In ten minutes after the bath, it fell, on one occasion, from fifty-seven degrees (to which the bath had reduced it from sixty-four degrees) to forty-five degrees."

A fourth source of error is the occurrence of shivering. In the course of the experiments above alluded to, I never failed to observe a striking change in the state of the circulation after the refrigerant process had been carried far enough to produce rigors, &c. To quote my own observations again, "On the supervention of shivering, I always found the pulse to rise in frequency, and become at the same time irregular, and rather difficult to count." A fifth source of fallacy is voluntary muscular exertion, whether immediately before the exposure or during the experiment: so powerful is the influence of muscular efforts in counteracting the sedative power of cold over the blood-vessels, that, under exposure to the most intense and freezing atmosphere pedestrian exercise is sufficient, in most instances, to preserve even the toes from asphyxia and congelation. A sixth fallacy arises from inattention to posture: it has been for a good many years known, and has lately been verified by some able inquirers of the Irish metropolis, that between the recumbent and erect postures there is a difference of more than a dozen beats per minute. A seventh source of error has been the not taking into account the stimulant operation on the heart of an unusual accumulation of blood in the great vessels, in consequence of refrigeration of the surface. Such are the circumstances, from inattention to which, as I believe, able writers have fallen into the error of attributing to cold a stimulant power over the heart and arteries.

Of the *sedative* power of cold over the circulation, we have interesting illustrations in the narratives of late travellers in polar regions. Captain Franklin mentions that anasarca was common and troublesome amongst his party during their excursions in the snowy regions of the Esquimaux. Captain Parry informs us, that the greatest difficulty was experienced in effecting the cicatrization of wounds and ulcers during his stay in winter quarters. During the late severe winter of 1029-30, I myself experienced the necessity of at least moderate warmth as a condition of healing superficial ulcers in the case of a young lady, who had scalded herself. Granulation was in this case very difficultly obtained, and cicatrization was not brought about without the aid of artificial warmth and unusual coverings.

Another effect of cold on the circulation is *turgescence*: a sharp blast, or the application of a very cold body to the skin, often produces vascularity, redness, and fulness. Extreme atmospherical cold operating on the whole surface of the body often produces priapism, or venous turgescence; and there is reason to believe that the spleen and other diverticula, and the great veins of the cavities, are similarly affected. But the turgescence of the arterial capillaries first mentioned, or reactive turgescence, otherwise vascular reaction, is probably not a direct or immediate effect of refrigeration, but rather the result of an erethism of the cutaneous nervules, occasioned by the sudden or very rapid abstraction of their caloric. The causation of the reactive efforts observed in the vascular system and calorific function, when subjected to refrigeration, will be hereafter considered.

But the morbific effect of cold on the circulation, which is perhaps the most important, is that change in the distribution of the blood which might be called *centralization,* and which consists in the retirement or abstraction of the fluids from the skin and superficial parts, and generally from the capillary arteries of the extremities, and their accumulation in the great trunks of the viscera and cavities, and principally, I imagine, in the great veins of the head, chest, and abdomen. This is an effect of protracted cold that is universally admitted, and highly probable, if not quite certain; but the necrotomic proofs are, it must be granted, rather scanty. Opportunities of examining the bodies of persons dead of protracted refrigeration are happily rare in this country. When death does happen from exposure, it is usually in cases in which exhaustion from labour and famine, and other circumstances, have conspired with cold, and the induction is consequently embarrassed. But there are several considerations that make for the conclusion generally adopted, and none that I have met with that have any weight in the other direction. Priapism is an effect of extreme atmospherical cold; now it is quite certain that the erectile tissues owe their occasional distention to venous congestion. Protracted immersion of the leg in very cold water generally produces a bluish redness of the skin, which is apparently a local venous congestion. The state of persons emerging from cold water, after the supervention of violent shivering, &c. resembles that of a sick man in the cold stage of fever, not only in the feeling of coldness and real deficiency of caloric, and in the muscular agitation and arterial disturbance, and contracted and functionally inert skin, &c., but also, in many cases, in the aching head, and oppressed chest and abdomen. I recollect very distinctly the appearance of a Genevese *commissionaire* who attended me some years since at Göttingen, and having often boasted of his skill in swimming, diving, &c., proceeded at length to give me a "taste of his quality." Having plunged from a height into a pond used by the students for bathing, he remained below, or at least out of sight of my friend Dr. Haycraft and myself, who watched him narrowly for about twelve minutes, and then ascended, or at least reappeared and came to land, having in many respects the appearance of a patient in malignant cholera. The pulse, indeed, was easily felt, but was weak, hurried, and very irregular; the face was hippocratic, with shrunken features and singularly wild and ghastly expression; the lips, extremities, &c. were purple or bluish; the skin of death-like coldness, &c.; he shivered violently, and complained of great inconvenience from oppression at the chest, &c., and assured me that he would never repeat the experiment: which I should mention he had voluntarily undertaken and gratuitously performed. As the story is singular, I may add that he represented himself as having acquired the power of enduring protracted immersion by diving in early youth for small pieces of money thrown by English and other foreign residents into Lake Leman; and that, on the particular occasion in question, he dived to the bottom, where, at a depth of about eighteen feet, he laid hold of a stone, and was thus enabled to maintain his position below.

The veins and the erectile tissues they supply readily admit of even great distention. The functions of the veins are principally passive; amongst several extrinsic forces, by aid of which they forward the blood towards the centre, is probably a pumping action of the right side of the heart. The arteries are elastic, and react with energy upon distending fluids, and readily shrink after the evacuation of their contents by hæmorrhage, or other mode of depletion. In the capillaries, the reactive force is probably very great. Those vessels, however, possess another property: they seem sensible to stimuli in the same manner nearly as muscular fibre. Amongst other stimuli, we are assured by Drs. Thomson and Hastings that a capillary has been seen to contract under the influence of ice, even so as to disappear; its contents having been expelled into the veins. These facts confirm the power of centralizing the blood above attributed to extreme cold. A refrigerated blood will stimulate the heart feebly, and that organ will consequently absorb less blood from the veins, and expel less into the arteries, than under other circumstances. The capillary arteries also, particularly the superficial ones, being torpified, and perhaps shrunk and rendered somewhat rigid by cold, will receive and forward less than their usual quantum. The spleen, the portal circle, the sinuses of the brain, and great pectoral vessels, are then the natural asyla or diverticula of the blood. The effects of heat upon refrigerated parts, or individuals, further confirm this position; which is of the utmost importance if well founded, and in favor of which, being well convinced that it is so, I am anxious to adduce every important fact and analogy I can find capable of, in any degree, compensating for the defect of necrotomic evidence. The changes in the sensation and in the external appearance of an individual refrigerated and shivering from a very cold atmosphere, all either favor the theory of centralization or are indifferent to it. The rapid return of lively sensibility, warmth, smoothness, fulness, redness, &c. to the skin upon the application of heat, all point to fluxion towards the surface and depletion of the asyla and diverticula above mentioned as their essential cause or necessary concomitant. The opinion under consideration is further corroborated by the great frequency of anal, pulmonary, and other internal hæmorrhages in cold climates and seasons: and there are other pathological facts that confirm it.

*Absorbents.* Absorption is retarded by cold seasons and atmospheres. Dropsies are difficult of cure, and frequent of occurrence in cold countries and seasons, not only because cold checks perspiration, and, by closing one great outlet, leaves much additional labour for other emunctories, but also because it diminishes the activity of the circulation, and checks those processes, chemical and mechanical, on the perfection of which depends a complete removal of effitte particles and effused fluids. If absorption be a vital process, it must obviously suffer from protracted refrigeration; and if it be physical or *endosmotic*, it is clear that it must stand in need of sufficient caloric as a necessary condition of fluidity, solubility, affinity, and perhaps permeability.

*Nervous System.* The morbid affections of the nervous system producible by cold are more numerous than those of any other system from the same agent; they comprise diseases, both positive and negative, of the sensitive nerves, of the motor nerves, and of the moral functions.

Cold produces both *pain* and *numbness:* of the former there are many varieties. There is the familiar disagreeable sensation, the feeling of chill or coldness, which is experienced in all degrees from mere coldness to a piercing, freezing cold. If our sensations were well watched, and as Macquart, I believe, truly says, "they are in such matters trustworthy and excellent monitors," we should, I am convinced, be able not only to escape the consequences of exposure on many occasions by timely retreat, or other precaution, but, when unavoidably or accidentally sufferers from the morbific power of cold, we should seldom be at any loss to identify the time and place of the injurious impression, and consequently should rarely be in any doubt as to the cause of our disorder. So sensitive is the nervous system in health to every movement of caloric, every fluctuation of temperature, that I am convinced that no dangerous refrigeration of the surface could pass unnoticed by those who attend to their sensations. Those impressions of cold that occasion diseases of internal organs by, as it would seem, some irritation transmitted from the surface, and which appear to be the usual occasions of rheumatism and of inflammation of the viscera, are commonly, if not invariably of this kind. The other forms of pain of cold usually attend or occasion inflammation of the tegument exposed. Pelham, in his account of the eight seamen left by accident at Spitzbergen, where they passed the winter of 1630, speaks of a *burning* pain: he informs us that, "after the new year, the frost became so intense as to blister their flesh like fire, and sometimes to seize them so that their flesh felt sore, as if they had been cruelly beaten." Another form of pain is that of *pricking, lancinating* pain which attends local asphyxia. MAUPERTUIS, in a passage above cited, informs us that he experienced a *lacerating* pain from inhalation of the cold air of a Tornea winter. MM. PARAT and MARTIN speak of similar forms of pain of cold. *Numbness* or *torpefaction* is a familiar effect of protracted or intense cold: it is rarely productive of important mischief. Incautious admission of heat to a part so far refrigerated as to have lost its sensibility is the usual occasion of morbid affections arising from the benumbing power of cold. In this way it is that chilblain, frostbite, and what has been very improperly called local asphyxia, are usually produced. Without reaction, turgescence, and inflammation, these consequences of exposure to cold are impossible; they are essentially phlogistic, and are the results of reaction occasioned generally by external heat, and always attributable to vehement reactive erethism of the cutaneous nerves, followed rapidly, sometimes almost instantaneously, by fluxion, tumefaction, vesication, gangrene, and sphacelus. The application of external warmth is, I have said, generally the occasion of this violent reaction. In rare cases, however, and commonly under the influence of intensely cold blasts operating on a vigorous frame, inflammation, vesication, &c. are observed to follow the exposure, independently of any subsequent external excitement. Numerous instances of this occur in the narrative of travellers and voyagers in arctic regions. Mr. PELHAM, already cited, mentions that the atmosphere of Spitzbergen sometimes blisters like fire. Captain MIDDLETON says, that "the fog and mists brought here" (Hudson's Bay,) from polar parts appear visible," &c.; "and if our hands or faces be uncovered, presently raise great blisters as white as a linen cloth and hard as horn." Of such effects erethism and turgescence, in a word reaction, is still the cause. The explanation is, I think, plainly this: instantaneous congelation of the cuticle follow the access of cold air; a warm, soft, pliant covering attached to a membrane most copiously supplied with sensitive nerves and capillary arteries, and consequently most delicate and susceptible, is all at once as it were petrified, and converted into a cold and rigid substance; and its attachments to the cutis are rudely strained and lacerated. To this mechanical injury if we add irritation of the nervules of the skin, from sudden transmission and escape of caloric, we shall have altogether sufficient materials for an explanation of the almost instantaneous turgescence and inflammation which must precede the effusion, vesication, &c. observed in such cases.

Cold is a common cause of paralytic, apoplectic, and convulsive seizures. Dr. Fothergill has remarked (and as a result of extensive and accurate observation, the remark would, even if unsupported by numerous other authorities, be entitled to great attention,) "that sudden transitions from heat to cold, and from cold to heat, often produce those complaints," (viz. apoplexy and palsy.) We are assured by Marcard and other writers, that the cold bath has often occasioned apoplectic and epileptic attacks.

The researches of Sir C. Bell have brought into additional notice a species of palsy before ill understood; namely palsy of the muscles of the mouth, cheek, &c. originating, in many cases, in exposure to cold, and depending on loss of functional power in what older anatomists called the portio dura of the seventh pair of nerves.

The disturbance of the higher cerebral functions produced by extreme atmospherical cold are various and striking. Insensibility, somnolency, delirium, maniacal fury, convulsive agitation, are the principal effects of intense cold on these functions. Several of these effects are strikingly exemplified in the following passage from Captain Parry's Third Voyage. In the eighth chapter of that interesting history, we are informed by Captain Lyon, that "several of his party" (they were on their return to the ships from an inland excursion in very severe weather, at a temperature below zero,) "began to exhibit symptoms of that horrid kind of insensibility which is the prelude to sleep; they all professed extreme willingness to do what they were told in order to keep in exercise, but none obeyed; on the contrary, they reeled about like drunken men; the faces of several were severely frost-bitten, and some had for a considerable time lost sensation in their fingers and toes: yet they made not the slightest exertion to rub the parts affected, and discontinued their general custom of warning each other on observing a discoloration of the skin." He afterwards adds, "my attention was particularly directed to Serjeant Spackman, who, having been repeatedly warned that his nose was frozen, paid no attention to it, owing to the state of stupefaction he had fallen into. The frostbite had now extended over one side of his face, which was frozen as hard as a mask; the eyelids were stiff, and one corner of the mouth was drawn so as to expose the gums and teeth. He complained sadly of giddiness and dimness of sight, and was so weak as to be unable to walk of himself." The following passage is still more striking. "Sometimes," says BEAUPRÉ, in his account of the Retreat from Moscow, (*Treatise on Cold*, chapter 4,) the eye was open, fixed, dull, wild, and the brain was seized by a quiet delirium; sometimes the eye was red, and announced transient excitement of the brain, and in those cases the delirium was better marked. Some stammered out incoherent words; others had a reserved and convulsive laugh; in some blood flowed from the nose and ears; they agitated their limbs, as if groping for something." "I have observed men overpowered by cold;" (he subjoins, still speaking of the sufferings of the French experienced under his own eyes in the vicinity of Smolensko): "I have seen them uncovering their breasts, agitating their arms as a man labouring under a deep delirium in an ataxic fever, in a state in which they certainly no longer felt desirous of food, &c." The irresistible propensity to sleep is very strikingly exemplified in the following anecdote related by the same writer of himself. "During the frightful night that we left Smolensko, I felt much harassed, and towards five in the morning a feeling of lassitude invited me to stop to rest. I sat down on the trunk of a birch tree, beside eight frozen corpses, and soon felt an inclination to sleep, to which I yielded, for it seemed delicious. I was fortunately dragged out of that incipient somnolency, which would infallibly have brought on torpor, by the cries and oaths of two soldiers opposite me, who were striking violently a poor exhausted horse that had just fallen down. I emerged from the state with a kind of shock; the sight of what was passing before me recalled strongly to my mind the danger to which I was exposing myself, &c." The illustrious BOERHAAVE, we are informed, I think, by Haller, narrowly escaped destruction from sleep and asphyxia during the winter of 1709.

(To be continued.) p. 198.

